# Non-Linear Conductivity Response of Graphene on Thin-Film PET Characterized by Transmission and Reflection Air-Plasma THz-TDS

**DOI:** 10.3390/s23073669

**Published:** 2023-03-31

**Authors:** Binbin Zhou, Mattias Rasmussen, Patrick Rebsdorf Whelan, Jie Ji, Abhay Shivayogimath, Peter Bøggild, Peter Uhd Jepsen

**Affiliations:** 1 Department of Electrical and Photonics Engineering, Technical University of Denmark, 2800 Kongens Lyngby, Denmark; 2 Department of Physics, Technical University of Denmark, 2800 Kongens Lyngby, Denmark

**Keywords:** graphene, AC conductivity, THz time-domain spectroscopy, reflection spectroscopy

## Abstract

We demonstrate that the conductivity of graphene on thin-film polymer substrates can be accurately determined by reflection-mode air-plasma-based THz time-domain spectroscopy (THz-TDS). The phase uncertainty issue associated with reflection measurements is discussed, and our implementation is validated by convincing agreement with graphene electrical properties extracted from more conventional transmission-mode measurements. Both the reflection and transmission THz-TDS measurements reveal strong non-linear and instantaneous conductivity depletion across an ultra-broad bandwidth (1–9 THz) under relatively high incident THz electrical field strengths (up to 1050 kV/cm).

## 1. Introduction

Graphene holds high promise as a key base material for next-generation electronics. In recent years, the development in graphene growth and transfer has been on the fast track, as exciting progresses such as chemical vapor deposition (CVD), graphene growth, and scalable roll-to-roll techniques have been achieved and are maturing [1,2,3]. For large-scale commercialization of graphene electronics, it is crucial to ensure graphene production with uniform and reproducible electrical properties. However, the development of techniques to measure the electrical properties of graphene is yet to catch up with the rapid progress of graphene production. Among different inspection tools, terahertz time-domain spectroscopy (THz-TDS) is capable of measuring key graphene electrical properties (such as conductivity, carrier scattering time, and mobility) in an important non-contact and non-destructive manner, and an inline THz scanner to accurately monitor large-scale graphene production is highly promising [4].

To date, most of the THz-TDS characterizations of graphene have been carried out in transmission mode (T-mode). For T-mode measurements, THz waves transmitted through graphene and its substrate are acquired. Various substrate materials have been applied, such as high-resistivity silicon (Si) [5,6,7,8], quartz [9], sapphire [10], and polymer films [11,12]. Because CVD graphene on polymer film is a versatile candidate for flexible or rigid touch screen displays, and also due to the fact that most graphene transfer methods rely on polymer as a handling layer, the ability to directly measure the electrical properties of a graphene sample on a thin-film polymer is of great practical significance. Quantitative THz-TDS characterization of graphene samples on the thin-film polymer is non-trivial due to the difficulty of separating the main THz waveform signal from the closely spaced and possibly temporally overlapping echo signals arising from the air/polymer interfaces [11]. It has been recently shown that the extremely short THz pulses generated from the two-color air-plasma process facilitate accurate determination of the graphene electrical properties of such thin-film samples due to easy echo separation and further aided by the broader obtainable conductivity spectral bandwidth [12]. Compared to T-mode THz-TDS, the reflection-mode (R-mode) technique is a good alternative, which offers more flexibility and is especially useful for highly absorptive samples/substrates. R-mode THz-TDS characterization of graphene electrical properties has only been reported on rigid substrates at relatively low THz frequencies [13,14,15,16]. Similar R-mode measurements of graphene on the technologically important flexible polymer substrates have so far not been reported.

Furthermore, due to its Dirac-type electronic band structure, graphene is a very non-linear material under the electrical field of THz radiation. Strong THz-induced transparency of CVD-grown graphene was reported with time-resolved THz pump/THz probe studies [17], as well as with non-linear THz-TDS investigations [18]. With an electrically gated sample, and thus a tunable graphene Fermi energy, the tuning of the graphene THz non-linearity was investigated with both quasi-monochromatic multicycle THz pulses and ultrashort single-cycle THz pulses [19] generated by optical rectification in lithium niobate crystals [20], covering the frequency range 0–2 THz.

In this report, we will show that the conductivity of graphene/thin-film polymer samples can be accurately measured with a reflection-mode air-plasma-based THz-TDS setup. The removal of the phase uncertainty associated with reflection-mode spectroscopy will be discussed. We will show that the ultra-broad spectral bandwidth can greatly improve the accuracy of such analysis and thus significantly suppresses the uncertainty often seen in standard reflection-mode measurements. The extracted graphene electrical properties are validated by transmission-mode measurements from the same sample. Both T-mode and R-mode measurements show strong non-linear and instantaneous conductivity depletion of graphene under relatively high incident THz electrical field strengths, representing the first non-linear graphene conductivity investigation with an ultra-broadband air-plasma-based THz source.

## 2. Materials and Methods

**Figure 1:**
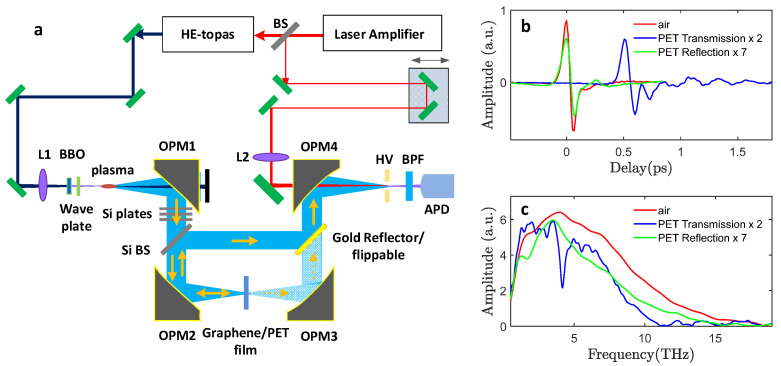
(**a**) A 1.4 µm laser driven two-color air-plasma-based THz-TDS which can easily switch between transmission-mode and reflection-mode configurations. L1, L2: lens with 300 mm focal length. HV: high voltage electrodes. BPF: band-pass filter at 400 nm. APD: avalanche photodiode. Si BS: silicon beamsplitter. (**b**) Typical THz waveforms of the original THz wave, the waveform transmitted through PET thin-film, and the measured waveform reflected from PET thin-film. (**c**) The corresponding spectra of the waveforms in (**b**).

The air-plasma-based THz-TDS setup is shown in Figure 1. To obtain high THz field strengths, laser pulses centered at a wavelength of 1.4 µm and 40 fs duration from a high-energy optical parametric amplifier (HE-TOPAS) pumped by a Ti: sapphire amplifier laser system (SpectraPhysics Spitfire Ace, 0.8 µm wavelength, 6 mJ pulse energy, 35 fs pulse duration, 1 kHz repetition rate) was used as the driving laser source [21]. A 100 µm thick BBO crystal was inserted for inline second harmonic (SH) generation after a lens with a 300 mm focal length. A dual-wavelength wave plate aligned the polarizations of the residual fundamental wavelength pulses and the SH wavelength pulses, ensuring efficient broadband THz wave emission with linear polarization. A small portion of the output directly from the laser amplifier was employed as the optical probe beam for the THz waveform detection. An 8 mm diameter hole in the center of the first off-axis parabolic mirror (OPM1, 4” effective focal length) eliminated most of the residual laser beam while reflecting most of the THz beam at the same time due to the conical emission pattern of the air-plasma THz wave emission [22,23]. Three off-axis parabolic mirrors (OPM2-4, all with 3” effective focal length) were employed to generate an intermediate focus on the sample, as well as the final focus at the high voltage (HV) electrodes (OPM3 was skipped for the R-mode configuration). A 20 THz long-pass filter (QMC Instruments) removed the residual pump laser light from the THz beam path. A 2 mm thick high-resistivity silicon plate was inserted into the collimated THz beam path before the sample at 45°, which picked up the reflection signal from the graphene/PET sample. Another 45° metallic reflector on a flip mount redirected the reflection signal into the air-biased-coherent-detection (ABCD) [24]. When the metallic reflector is flipped down, the setup is switched into standard T-mode measurements, with the exact same sample position as the R-mode measurement. Multiple (up to 10) high-resistivity silicon wafers with minimal dispersion and absorption in broadband THz frequency range were used to adjust the incident THz pulse field strength onto the graphene sample [25] according to the attenuation formula $E_{N}={\left(0.7\right)}^{N}$, where *N* is the number of wafers inserted in the beam. With zero attenuation wafers, the THz pulse energy was 0.51 µJ at the sample location, and the calculated peak THz electrical field strength was 1050 kV/cm, based on measurement of the beam profile and the time trace detected by ABCD [23]. The incident THz field can be reduced with the insertion of wafers to a minimum of 30 kV/cm with 10 wafers inserted. The setup was purged with pure nitrogen to avoid the influence of water vapor absorption.

**Figure 2:**
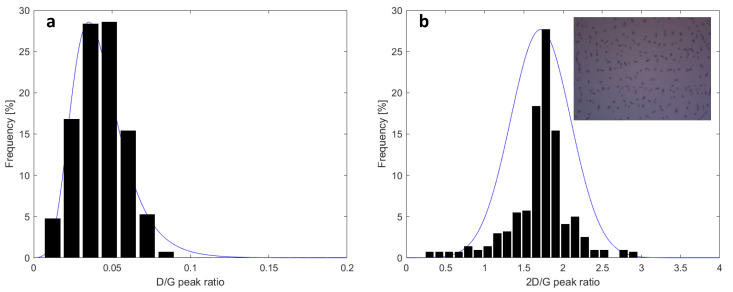
(**a**) The Raman histogram of the ratio of D band to G band intensity. (**b**) The Raman histogram of the ratio of the 2D band to G band intensity. Inset, an optical image of the graphene.

The graphene was grown on copper foil by chemical vapor deposition. The as-grown graphene on copper was laminated on a thermal release tape and then transferred onto polyethylene terephthalate (PET) film with a thickness of 230 µm. The PET film was partially covered by graphene, and the bare film surface served as a reference for the THz-TDS measurements. Prior to THz measurements, Raman spectroscopy was employed to check the quality of the graphene sample. Due to the fact that organic PET substrates tend to heavily pollute the Raman signal from graphene, Raman characterizations were performed after transferring the graphene onto a high-resistivity silicon substrate with a 90 nm thin oxidized surface layer (SiO_2_). The results are shown in Figure 2. Figure 2a shows the Raman histogram of the D band to G band intensity ratio. A median value of 0.04 indicates that graphene contains very few defects in its atomic structures [26]. The 2D/G Raman histogram (Figure 2b) points to a median value of 1.75, indicating the graphene sample is mostly in the single-layer form [27]. The single-layer graphene is also verified by the dominant background color in the optical image taken from the same graphene-on-Si/SiO_2_ sample (inset in Figure 2b). The black lines on the optical image are wrinkles from the transfer process, whereas the darker spots are bi- or more-layer graphene around the nucleation spots where the growth was initiated on the Cu catalyst [28].

The measured THz waveform and spectrum without insertion of samples (air transmission) are shown as the red curves in Figure 1b,c. The waveform transmitted through the 230 µm PET is delayed by approximately 0.5 ps (blue curve) and shows significant attenuation due to absorption loss. Besides a distinctive, narrow absorption band at 4.2 THz, there is a rather strong absorption for high frequencies above 10 THz. The measured reflection waveform (green curve) is rather weak compared to the transmission signal, primarily due to the relatively small refractive index of PET (n ≈ 1.7). Despite the relatively low amplitude, the reflection signal has a significantly smoother and broader spectrum, which is advantageous for spectroscopic characterizations. We note that the reflection THz waveform experiences further attenuation from the additional reflection on the silicon beamsplitter before entering the waveform detection, leading to the reflection signal being significantly weaker than the transmission signal in the waveform detection process.

## 3. Results and Discussion

### 3.1. Measurements in Transmission-Mode Configuration

In order to benchmark the reflection-mode characterization, the sheet conductivity of the graphene/thin-film PET sample was first characterized with standard transmission-mode THz-TDS. The transmitted THz waveforms through the bare PET substrate and through the graphene-covered substrate were sequentially obtained for the extraction of the graphene electrical properties as functions of the frequency ω=2πν. Subsequently, ${\tilde{E}}_{\mathrm{film}}\left(\omega \right)$ and ${\tilde{E}}_{\mathrm{sub}}\left(\omega \right)$ can be obtained as the Fourier transforms of the pair of measured THz waveforms. The transmission function ${\tilde{T}}_{\mathrm{film}}\left(\omega \right)={\tilde{E}}_{\mathrm{film}}\left(\omega \right)/{\tilde{E}}_{\mathrm{sub}}\left(\omega \right)$ is then used to calculate the frequency-dependent sheet conductivity of graphene, $\sigma_{s}\left(\omega \right)=\sigma_{R}\left(\omega \right)+i\sigma_{I}\left(\omega \right)$. For graphene/thin-film substrate samples, it is usually tricky to separate the direct transmission (or reflection) waveform from the closely following echo signals, since the temporal separation is similar to, or even shorter than, the THz pulse duration of a commonly used commercial THz-TDS system. For the air-plasma-based THz-TDS, the THz pulse duration is extremely short, such that echo signals originating from the PET/air interfaces can be easily separated from the directly transmitted transient, and $\sigma_{s}\left(\omega \right)$ can be determined straightforwardly as $\sigma_{s}\left(\omega \right)=\left(1/{\tilde{T}}_{\mathrm{film}}\left(\omega \right)-1\right)\left(n_{\mathrm{sub}}-1\right)/Z_{0}$, with $n_{\mathrm{sub}}$ being the substrate refractive index, and $Z_{0}=377\;\Omega$ is the vacuum impedance.

**Figure 3:**
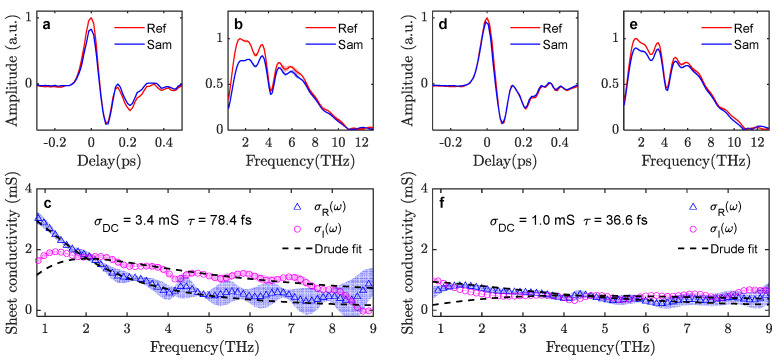
(**a,b**) Transmitted waveforms and spectra through bare PET (Ref) or through graphene/PET (Sam) with an incident THz field strength of 60 kV/cm. Light red and blue bands indicate the experimental standard deviation between scans. (**c**) Extracted complex-valued conductance spectrum of the graphene under low THz field strength (together with the Drude model fitting curves). Light blue and magenta bands indicate the standard deviation of the calculated conductance. (**d,e**) Transmission waveforms and spectra (Ref and Sam) with an incident THz field strength of 1050 kV/cm. (**f**) Extracted and fitted conductance spectra at strong THz field strength.

Transmission-mode measurements at the low THz field (N=8 Si wafers, 60 kV/cm incident THz field strength) as well as at the strongest THz field (N=0 Si wafers, 1050 kV/cm field strength) are shown in Figure 3. Each set of measurements is based on two reference scans and two sample scans. Light color bands indicate the standard deviation of the measurements (or the standard deviation of the calculated sheet conductivity). At low THz field strength, the absorption in the graphene film is significant, with up to 18% attenuation of the incident waveform. The absorption is frequency dependent, with stronger absorption at lower frequencies.

**Figure 4:**
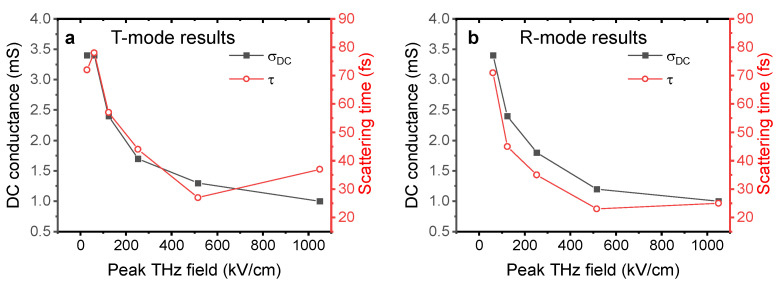
(**a**) DC conductance and scattering time obtained from Drude fits at various incident peak THz field strengths from the transmission-mode THz-TDS measurements. Solid squares: DC conductance. Open circles: scattering time. (**b**) The same parameters extracted from Drude fits reflection-mode measurements.

The extracted conductance spectra can be well fitted with the Drude model, $\sigma_{s}\left(\omega \right)=\sigma_{\mathrm{DC}}/\left(1-i\omega \tau \right)$, where $\sigma_{\mathrm{DC}}$ and τ is the DC conductance and electron scattering time, respectfully. This indicates a high-quality graphene sample where free carrier scattering is isotropic without significant influence of backscattering from extended defects such as grain boundaries. The Drude model fit across the 1–9 THz range agrees well with the extracted complex-valued conductance spectrum from the measured data and yields an estimated DC conductance of 3.4 mS and a scattering time of 78.4 fs. In contrast, at the maximum incident THz electrical field, THz wave absorption by the graphene film was strongly suppressed (the incident THz waveform attenuation is down to a maximum of 6.6%). The extracted complex conductance spectrum can still be fitted by the Drude model (shown in Figure 3f), however, with a significantly reduced DC conductance, $\sigma_{\mathrm{DC}}=1.0$ mS, and scattering time, τ=36.6 fs. Measurements with attenuation from different numbers of Si wafers were also implemented. A summary of the extracted graphene electrical properties at various incident THz field strengths is shown in Figure 4a. With 10 pieces of Si wafer attenuation (30 kV/cm), $\sigma_{\mathrm{DC}}=3.4$ mS and τ=72 fs are obtained from the fit, which indicates that the onset of the non-linear graphene conductivity response happens when the peak THz field strength is approximately 60 kV/cm. With further increase in the incident THz field, both the DC conductance and carrier scattering time decrease, and the depletion of the conductivity gradually saturates. It is worth noting that, similarly to previously reported CVD graphene non-linear THz-TDS investigations [18,19], the measured THz waveform at an instant *t* represents the interaction between the incident THz electrical field at *t* and the state of the graphene by the accumulated excitation of THz electrical field from the pulse start $t_{0}$ until right before *t*. Since the THz pulses here are extremely short (a few 10 s of fs), the strong non-linear response observed in this investigation indicates that the graphene electronic state can be non-linearly modulated by a strong transient electrical field in a time scale as short as few 10 s of femtoseconds.

### 3.2. Phase Corrections in Reflection-Mode Air-Plasma THz-TDS

Reflection geometry THz-TDS offers unique advantages in many spectroscopic application scenarios. However, there is one major obstacle that is commonly associated with reflection-mode THz spectroscopy: the phase difference uncertainty between the reference and sample waveform measurements due to small but in practice unavoidable errors in positioning of the sample reflection plane with respect to the reference reflection plane. Similar to T-mode THz spectroscopy, the reflection function ${\tilde{R}}_{\mathrm{film}}\left(\omega \right)={\tilde{E}}_{\mathrm{film}}\left(\omega \right)/{\tilde{E}}_{\mathrm{sub}}\left(\omega \right)$ is necessary to calculate the graphene conductance $\sigma_{s}\left(\omega \right)$ in the R-mode measurements. In order to obtain ${\tilde{E}}_{\mathrm{film}}\left(\omega \right)$ and ${\tilde{E}}_{\mathrm{sub}}\left(\omega \right)$, waveforms reflected from the bare substrate surface and from the graphene-covered surface need to be measured in turn. In the T-mode configuration, shifting between reference and sample would normally not lead to unintentional phase differences. In contrast, for R-mode configuration, even minor displacement of the reflection plane due to unintended shifts or tilts of the sample surface would lead to an artificial phase offset δϕ=ωΔt=2ωΔd/c, where Δt is the time delay due to the unintentional shift in position Δd of the reflection plane and *c* is the speed of light. Since the actual physical phase shift due to the graphene-THz response is rather small, a tiny amount of δϕ can easily be dominating. Such small phase offset uncertainty is very difficult to avoid, and even very small displacements in the range of tens of nm can bring strong distortions and uncertainties to the material property extraction of the THz-TDS measurements.

To tackle the phase offset uncertainty issues, a great deal of effort has been made in the THz spectroscopy community. For instance, Pashkin et al. designed a special THz reflection spectrometer where the optical probe and THz beams maintain the same beam path lengths and thus introduce minimal phase shift uncertainty [29]. Window materials with precisely known refractive indices and thicknesses were utilized for taking reference and sample waveform measurements at front and back interfaces, thereby minimizing the phase uncertainty [30]. The numerical maximum entropy method for artificial phase offset correction has been demonstrated [31]. By simultaneous measurements of two orthogonal components of the reflected THz electrical fields, the phase uncertainty can be minimized in THz time-domain spectroscopic ellipsometry applications [32,33]. For highly doped semiconductor materials whose electrical properties can be well described by certain response models, subtraction of an artificial linear phase shift for optimum model fitting has also been reported [34].

When translating between the bare substrate surface and the graphene-covered surface on the large-size CVD graphene/thin-film PET samples, the artificial time delays, Δt, can, due to the displacement of the reflection planes, reach up to 200 fs (corresponding to a shift of the reflection planes Δd = 30 µm). On the other hand, the actual physical phase change of the incident THz waveform induced by the graphene is much smaller. Without proper correction for the artificial phase difference, the calculation of the graphene conductance from the reflection function ${\tilde{R}}_{\mathrm{film}}\left(\omega \right)$ would be faulty. The artificial phase offset is strictly linear with respect to frequency, whereas the phase shift between sample and reference due to the frequency-dependent conductance is not a linear function of frequency. Therefore, numerical removal of the linear slope on the phase difference curve (which is equivalent to lining up the two temporal waveforms) can significantly improve the graphene conductance retrieval. In practice, there is still a certain amount of uncertainty with the linear phase slope removal. For materials with certain response models (e.g., Drude, Drude–Smith, or Lorentzian), the remaining phase difference uncertainly can be further reduced in an automated and quantitative manner by fine-tuning the phase offset correction for optimum model fitting. We will show in the following how a known fitting model (the Drude model in this case) can be utilized for accurate artificial phase offset correction in the reflection-mode air-plasma THz-TDS and subsequent extraction of graphene electrical properties.

**Figure 5:**
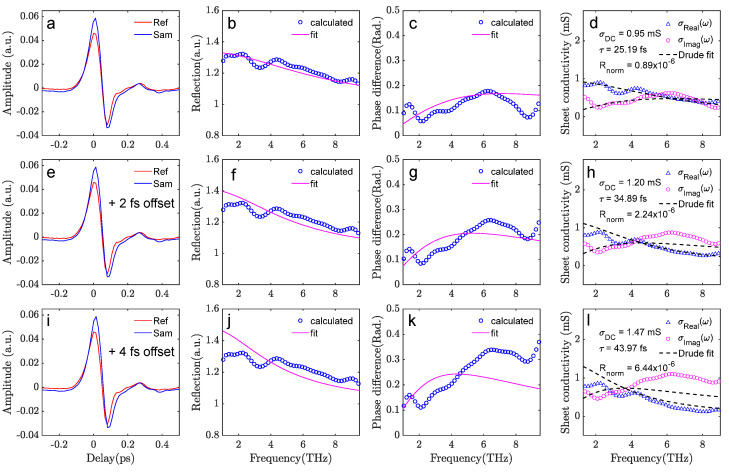
Phase offset correction of the reflection-mode THz-TDS data analysis. (**a–d**) With optimum offset correction, the extracted conductance spectra match the best with the Drude model. The calculated reflection and phase difference curves also match best with the fit. (**e–h**) With 2 fs extra phase offset, the extracted parameters show significant deviation from the Drude model fits. (**i–l**) With 4 fs extra offset, a Drude fit is impossible in the broad frequency range.

The red curve (Ref) in Figure 5a represents the measured reflected waveform from the PET, and the blue (Sam) is the reflection from graphene/PET. Due to the existence of free carriers in the graphene sheet, the sample signal is significantly higher in amplitude. The as-measured reference and sample waveforms show an artificially large time delay (phase offset). For optimum Drude model fitting, a time delay Δt = 149 fs has already been numerically subtracted in Figure 5a. With this phase correction, the complex graphene conductance spectrum is extracted together with the optimum Drude model fitting curves at a broad frequency range (1.5–9 THz), as shown in Figure 5d. There is good agreement between the measured data and the fit, and the squared norm of the residual $R_{\mathrm{norm}}=0.86\times 10^{-6}$ from the fit represents the minimum $R_{\mathrm{norm}}$ when fine-tuning the phase slope for optimum phase offset correction. The calculated phase difference and reflection curves, together with the calculated value from the Drude fit parameters, are shown to be in good agreement in Figure 5b,c. To demonstrate the precision of the phase correction procedure, Figure 5e–h shows the results when only 2 fs of extra shift is introduced to the optimum correction case. The calculated graphene conductance spectrum leads to a significantly worse Drude fit, with the fitting $R_{\mathrm{norm}}=2.24\times 10^{-6}$, which is 2.5 times higher compared to the optimum case. Deviations between the calculated and fitted reflection and phase difference curves are clearly visible. Figure 5i–l shows the situation of 4 fs of extra time delay shift, and it is evident that the extracted graphene electrical properties are now clearly non-Drude in the 1.5–9 THz range. It is worth noting that the ultra-broad bandwidth available in this study is essential for the very fine delay/phase correction; the uncertainty from the described correction procedure will be greatly compromised with a narrower spectral bandwidth.

### 3.3. Measurements from the Reflection-Mode Air-Plasma THz-TDS

**Figure 6:**
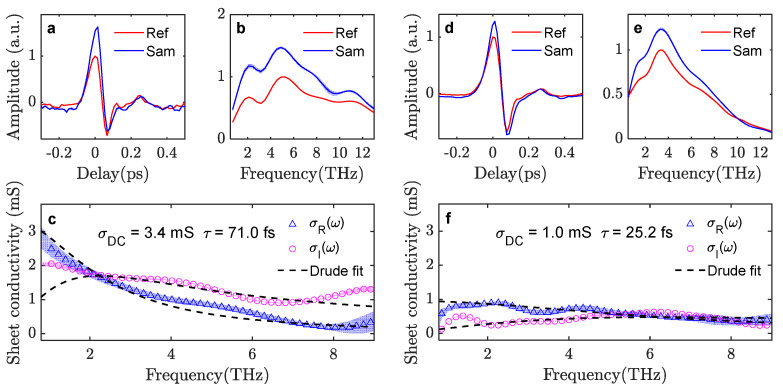
Reflection-mode measurement results. (**a,b**) Reflection waveform and spectra through bare PET (Ref) or through graphene/PET (Sam) in the incident THz field strength of 60 kV/cm. Light red and blue bands indicate the experimental standard deviation between scans. (**c**) Extracted complex-valued conductance spectrum of the graphene under low THz field strength (together with the Drude model fitting curves). Light blue and magenta bands indicate the standard deviation of the calculated conductance. (**d,e**) Reflection waveform and spectra (Ref and Sam) with the incident THz field strength of 1050 kV/cm. (**f**) Extracted and fitted conductance spectra under strong THz field strength.

By implementing the above-mentioned phase correction procedure, graphene properties can be extracted from the R-mode THz-TDS measurements. We show the results in Figure 6 for the case of the low incident THz field (60 kV/cm) as well as the strong THz field (1050 kV/cm). R-mode measurements at 30 kV/cm incident field are not available as the THz signal strength in the ABCD detection is too low for reliable detection (due to the extra attenuation in R-mode as mentioned earlier). At low THz field strength, the conductivity of graphene results in a much stronger reflection signal compared to the reflection signal from bare PET, and the waveform amplitude is up to 63% stronger. The extracted $\sigma_{\mathrm{DC}}=3.4$ mS, and scattering time τ=71.0 fs, agree well with the parameters extracted from the T-mode measurements under the same field strength. At 1050 kV/cm field strength, the contrast between the reference and sample waveform amplitudes is reduced to a maximum of 28%, indicative of a strongly suppressed graphene conductivity. The extracted $\sigma_{\mathrm{DC}}=1.0$ mS is the same as the value extracted from T-mode measurements. The extracted scattering time, τ=25 fs, is, however, somewhat smaller than the 37 fs scattering time extracted from the T-mode. The overview of the graphene electrical properties as a function of THz field strength, as extracted from R-mode THz-TDS, is shown in Figure 4b for direct comparison with the T-mode measurements. The extracted $\sigma_{\mathrm{DC}}$ and τ from the T-mode and R-mode measurements agree well in most of the cases, which validates the phase correction procedures applied to the R-mode TDS analysis in this work. We have shown here that the procedure applies to high-quality continuous graphene samples with a Drude-like conductivity response, but we note that the procedure should also be applicable to other types of thin-film materials with other conductivity response functions. The only discrepancy is the high τ in the T-mode measurement under the strongest THz field strength of 1050 kV/cm.

It is evident that, compared to the T-mode, the same graphene film leads to a much larger Ref-Sam contrast in the reflection-mode measurements. In the 1050 kV/cm case when the graphene conductivity is significantly suppressed, the R-mode measurements still have a reference-sample contrast of a maximum of 28%, whereas it is only 7% from the T-mode measurements. It is reasonable to assume that the much larger signal contrast in the R-mode would lead to a more convincing extraction.

It is worth noting that the observed peak THz field strength for the onset of non-linear graphene conductivity responses in this study (approximately 60 kV/cm) is higher than what was reported in previous studies (e.g., 20 kV/cm in Ref. [19]). This is most likely due to the different shapes of the THz pulses applied for driving the graphene non-linear process in those experiments. In previous reports (Refs. [17,18,19]), THz pulses generated by optical rectification in lithium niobate crystals were employed, which have sub-ps pulse duration. In this study, the THz pulses from the two-color air plasma have durations of only a few tens of fs, which is at least one order of magnitude shorter. Although the observed graphene non-linear response driven by sub-ps THz pulses can be quantitatively explained by a thermodynamic model that is validated for time scales longer than the electron scattering time in graphene, the non-linear response of graphene to air-plasma THz pulses may be quite different from such a relatively slow mechanism. The non-linear physics behind the faster non-linear conductance dynamics observed here is currently under investigation and is outside the scope of this work.

## 4. Conclusions

To summarize, we report on the first sheet conductivity measurements of graphene on thin-film polymer samples with reflection-mode THz time-domain spectroscopy. The correction of the phase uncertainty associated with reflection-mode spectroscopy is discussed, and it is shown that the ultra-broad bandwidth available from the air-plasma-based THz-TDS significantly improves the accuracy of phase correction and enables the quantitative extraction of the DC conductance and electron scattering time of graphene. The extracted graphene electrical properties are directly compared with transmission-mode measurements from the same sample. Both the T-mode and R-mode measurements show strong non-linear conductivity suppression of graphene under high incident THz electrical field strengths, which represents the first non-linear graphene conductivity investigation with an ultra-broadband air-plasma-based THz source. Due to the much faster time scale involved, the reported graphene non-linear conductivity response is different from the thermodynamic non-linearity observed under the excitation of sub-ps THz pulses.

## Data Availability

The datasets analyzed or generated during the study are available from the corresponding author on reasonable request.
